# Meeting of American Graduates at Sydney, New South Wales

**Published:** 1900-03

**Authors:** H. Taylor

**Affiliations:** Honorary Secretary


### Obituary.

### MEETING OF AMERICAN GRADUATES AT SYDNEY, NEW SOUTH WALES.

A meeting of American graduates resident in New South Wales was held at the offices of Dr. E. R. Magnus, Liverpool Street, Sydney, on November 20 last, to express their feeling of esteem in which the late Dr. Bonwill was held amongst them.

Dr. Alfred Burne occupied the chair, and amongst those present were Dr. E. Randolph Magnus, Dr. Frank Magnus, Dr. Carter, Dr. Arthur Cox, Dr. Oscar Davis, Dr. Hinder, Dr. Stanley Rea, Dr. Nathan, Dr. MàcTaggart, and others. Apologies were received from many unable to attend, owing to distance and short notice of meeting.

The following resolution was put and carried unanimously:

"That we, American graduates resident in New South Wales, desire to place on record our feeling of the great loss sustained by the dental profession through the untimely death of Dr. W. G. A. Bonwill, whose good work will long live to testify to his great ability, and his name will ever remain a landmark in the history of dentistry. We also desire to express our deep sympathy with those members of his family who are left to mourn their loss."

Dr. Burne in feeling words spoke of the many acts of kindness shown him by the late Dr. Bonwill, and also of the great loss that dentistry had suffered in losing such a skilful dentist, and in conjunction with the late Dr. Garretson, two of the most brilliant lights in the dental world. He felt that every graduate must well remember the clinics given by the late Doctor, which (speaking personally) will remain ever green in his memory.

Dr. Hinder, in moving the resolution, was unable to express his sympathy in fitting terms for the great loss sustained. Dr. Nathan seconded the resolution. Dr. Arthur Cox, Dr. Stanley Rea, and Dr. E. R. Magnus also spoke of the many personal acts of kindness received from the Doctor.

A vote of thanks to the chairman closed the meeting.

H. TAYLOR,
*Honorary Secretary.*

